# Eccentric Hamstring Strength and Interlimb Asymmetry in Professional Football Players: A NordBord-Based Longitudinal Analysis of Two Professional Teams

**DOI:** 10.3390/life16030532

**Published:** 2026-03-23

**Authors:** Tolga Altuğ, Mehmet Söyler, Coşkun Yılmaz, Meriç Eraslan, Ahmet Serhat Aydın, Mustafa Nurullah Kadı, Pelin Akyol, Hamza Küçük

**Affiliations:** 1 Faculty of Sports Sciences, Agri Ibrahim Cecen University, Agri 04100, Türkiye; taltug@agri.edu.tr; 2 Vocational School of Social Sciences, Cankiri Karatekin University, Cankiri 18200, Türkiye; mehmetsoyler@karatekin.edu.tr; 3 Kelkit Aydın Doğan Vocational School, Gumushane University, Gumushane 29100, Türkiye; 4 Faculty of Sport Sciences, Akdeniz University, Antalya 07058, Türkiye; mericeraslan@akdeniz.edu.tr; 5 Institute of Health Sciences, Gazi University, Ankara 06500, Türkiye; serhataydingazi@gmail.com (A.S.A.); nrllh.291@gmail.com (M.N.K.); 6 Yasar Dogu Faculty of Sport Sciences, Ondokuz Mayis University, Samsun 55270, Türkiye; pakyol@omu.edu.tr

**Keywords:** biomechanics, eccentric muscle function, hamstring strain injury, interlimb asymmetry, nordic hamstring exercise

## Abstract

The aim of this study was to examine temporal changes in eccentric hamstring strength, impulse-based mechanical outputs, and interlimb asymmetry in professional football players performing a football-specific eccentric hamstring training program. Forty male football players (18–25 years) from two teams competing in the Turkish Second Professional Football League participated in this longitudinal cohort study. Eccentric hamstring performance was assessed at three time points (pre-, mid-, and post-season) using the NordBord Hamstring Testing System. Mixed-design repeated-measures ANOVA revealed significant main effects of time and significant time × Team interactions for left and right maximal impulse values (*p* < 0.05). In contrast, maximal eccentric force variables showed no significant time effects, although significant time × Team interactions were observed for both limbs (*p* < 0.05). Interlimb maximal force asymmetry and mean asymmetry demonstrated significant time effects, while Team effects and interaction terms were not significant. Overall, these findings indicate that temporal changes in eccentric hamstring performance in professional football players may be more clearly reflected in force–time–dependent metrics, particularly impulse, rather than peak force outputs. Accordingly, impulse-based measures may provide additional insight into eccentric hamstring performance changes in professional football players.

## 1. Introduction

The marked increase in game tempo observed in modern football over recent years has fundamentally transformed both the quantity and quality of performance related physical demands during match play [1]. In particular, the growing frequency of high intensity running, repeated sprint efforts, and rapid acceleration and deceleration cycles has resulted in substantially greater mechanical loads being imposed on the muscles and tendons of the lower extremities within increasingly shorter time frames [2,3]. Within this context, the hamstring muscle group has emerged as one of the most critical structures in modern football with respect to injury risk, owing not only to its essential role in sprint performance but also to its function under high speed and eccentric contraction conditions [4,5].

Hamstring strain injuries remain among the most prevalent muscle injuries in professional football and, due to their high recurrence rates, continue to impose a considerable burden on performance continuity throughout the competitive season [6]. Long term epidemiological data indicate that no meaningful reduction in hamstring strain injury incidence has been achieved in recent years and that, at the elite level, these injuries account for a substantial proportion of total training and match related time loss [7]. This persistent trend highlights a critical issue. Beyond the implementation of preventive training strategies, it is equally important to determine how the neuromuscular and mechanical adaptations induced by these strategies are evaluated and quantified [8].

From a biomechanical perspective, the majority of hamstring injuries have been reported to occur during the terminal swing phase of sprint running. During this phase, the hamstring muscles act eccentrically to decelerate knee extension while simultaneously operating under high tension to counter hip flexion [9]. These mechanical conditions, which are characterized by rapid lengthening of the muscle tendon unit alongside high force production, challenge structural tolerance and increase the risk of tissue damage when eccentric capacity is insufficient [10,11]. Consequently, eccentric performance characteristics occupy a central role in the assessment of hamstring function [12].

Interventions aimed at enhancing eccentric hamstring capacity commonly include Nordic hamstring exercise-based protocols, which are widely recommended on the basis of strong epidemiological evidence [13]. Meta analytic findings have demonstrated that Nordic hamstring exercise implementation can substantially reduce the risk of hamstring strain injuries [14]. Nevertheless, the specific mechanical adaptations underlying this protective effect, and the performance metrics that most sensitively capture these adaptations, remain insufficiently clarified [15]. Most existing studies have evaluated eccentric adaptations primarily through maximal force or peak torque values. However, such metrics provide only a limited representation of the force time behavior of the muscle throughout the eccentric phase [16].

In functional tasks such as sprinting, the defining feature of eccentric muscle function is not solely the magnitude of peak force, but rather the ability to sustain force production across the eccentric phase and dissipate mechanical energy effectively [17]. Within this framework, impulse, which is defined as the time integral of force produced during the eccentric phase, offers a more holistic indicator of the muscle load tolerance capacity [18]. Mean force represents a complementary phase sensitive output that reflects the continuity of force production throughout the eccentric phase. These metrics may reveal adaptation patterns that are not captured by peak force alone, particularly under conditions of repeated sprinting and fatigue [19].

Alongside these considerations, interlimb force asymmetry has emerged as an important component in the evaluation of eccentric hamstring function [20]. Although strength imbalances among football players have been associated with hamstring strain injury risk, it remains unclear how this relationship varies across different mechanical outputs such as maximal force, mean force, or impulse [21]. Evidence that some athletes exhibit pronounced asymmetries in phase-based metrics despite minimal peak force asymmetry suggests that interlimb asymmetry may be a parameter sensitive and dynamic characteristic rather than a fixed trait [22].

Among field-based assessment tools, the NordBord system enables reliable bilateral measurement of eccentric knee flexor performance, providing data on both force magnitude and force time characteristics [23]. However, the existing NordBord literature has predominantly focused on peak force outputs, while phase sensitive metrics such as impulse and mean force, as well as their associated asymmetry profiles, have received limited attention. This limits a comprehensive understanding of the metric dependent nature of eccentric hamstring adaptations.

Therefore, the purpose of the present study was to examine the temporal effects of a football specific eccentric hamstring training model implemented in professional football players on multiple mechanical performance outputs, including maximal force, maximal torque, mean force, and impulse. It was hypothesized that eccentric adaptations would be more prominently reflected in phase sensitive metrics independently of peak force, and that interlimb asymmetry would demonstrate distinct response patterns depending on the mechanical parameter assessed. This approach aims to provide a more sensitive and multidimensional monitoring perspective for the evaluation of eccentric hamstring function.

## 2. Materials and Methods

### 2.1. Research Design

This study was designed as a longitudinal cohort study with a pre-test–mid-test–post-test design to examine temporal changes in eccentric hamstring muscle strength and interlimb force asymmetry in professional football players. The same football-specific eccentric hamstring training model was applied to two professional football teams, and measurement values obtained at three time points (pre-test, mid-test, and post-test) were compared. All assessments were conducted on the teams’ natural grass pitches under similar environmental conditions, using identical measurement equipment and standardized testing protocols, with the aim of minimizing measurement-related bias. Accordingly, any observed differences in eccentric hamstring strength and interlimb asymmetry were interpreted as reflecting team-related physical and neuromuscular characteristics observed across the monitoring period.

### 2.2. Participants

An a priori power analysis performed with G*Power 3.1 indicated that a minimum total sample size of 36 participants was required for a mixed-design repeated-measures ANOVA (f = 0.25, α = 0.05, power = 0.90, two groups, three measurements). Accordingly, the final sample of 40 players was considered adequate for the planned analyses. A total of 40 male football players volunteered to participate in the study, comprising Team A (Antalyaspor; *n* = 20) and Team B (Ankaragücü; *n* = 20), drawn from two teams competing in the Turkish Football Federation (TFF) Second Professional Football League. Team classification was based on team membership, with each team representing one professional football squad. Participants were between 18 and 25 years of age and were required to have at least five years of regular competitive match experience. Inclusion criteria were defined as the absence of any musculoskeletal injury or surgical history affecting the lower extremities within the previous six months and the continuation of regular training and match participation throughout the measurement period. Exclusion criteria included a history of acute or chronic musculoskeletal injury involving the lower extremities (muscle, tendon, ligament, or joint-related), previous lower-extremity surgery, the presence of any neurological, cardiovascular, metabolic, or orthopedic condition that could affect test performance, and inability to complete the testing protocol due to pain or discomfort during the measurement or training process [24]. All 40 players completed the testing sessions throughout the study period, and no missing data were observed. The mean age of the participants was 20.62 ± 0.23 years for Team A and 21.23 ± 0.44 years for Team B. The demographic characteristics of the participants are presented in Table 1.

### 2.3. Assessment of Eccentric Hamstring Muscle Strength

Eccentric hamstring muscle strength was assessed using the NordBord Hamstring Testing System (Vald Performance, Brisbane Australia). This system is capable of measuring, analyzing, and automatically recording eccentric force produced by the knee flexor muscles during the Nordic hamstring exercise for each lower extremity separately, with data expressed in Newtons (N). By simultaneously recording force data from the right and left lower extremities within the same movement cycle, the system allows for the assessment of bilateral force differences and interlimb asymmetry. The validity and reliability of NordBord-derived eccentric hamstring strength measurements have been demonstrated in previous research, with intraclass correlation coefficients (ICC) typically ranging between 0.83 and 0.90 and coefficients of variation reported between approximately 6% and 9% in athletic populations [16]. All measurements were conducted on the teams’ natural grass pitches under standardized field conditions, using the same equipment and testing protocols to enhance measurement reliability and minimize the influence of external variables.

During each testing session, players performed three maximal Nordic hamstring trials, and the highest valid force value obtained from the trials was used for statistical analysis. Interlimb asymmetry was calculated using the following formula:

Asymmetry (%) = (|Right − Left|/max (Right, Left)) × 100.

### 2.4. Outcome Definitions

Peak eccentric hamstring force was defined as the highest force value recorded during the eccentric phase of the Nordic hamstring exercise. Mean eccentric force represented the average force generated throughout the eccentric phase of the movement. Impulse was calculated as the integral of the force–time curve during the eccentric phase of the Nordic hamstring exercise, defined as the period from the initiation of forward trunk movement until the participant could no longer resist the forward descent. Impulse values were expressed in Newton-seconds (N·s). Hamstring torque values were estimated by multiplying the measured force output by the assumed knee joint moment arm corresponding to the distance between the knee joint center and the ankle fixation point of the device, with results expressed in Newton-meters (N·m).

### 2.5. Warm-Up Protocol

Prior to all testing sessions, a standardized dynamic warm-up protocol consisting of three stages was implemented to prepare the lower-extremity musculoskeletal system for testing conditions and to minimize the risk of potential injury. The detailed structure and content of the warm-up protocol are presented in Table 2.

### 2.6. Nordic Hamstring Test Application and Measurement Procedure

During test measurements, all players were provided with a single demonstration to ensure familiarity with the device and to enable them to maintain the manufacturer-recommended standard testing position. Participants were positioned on the NordBord Hamstring Testing System in accordance with the standard positioning protocol specified by the manufacturer; the trunk, hip, and knee joints were maintained in neutral alignment to minimize compensatory movements and postural alterations that could occur during testing. During the test, players were instructed to lean the trunk forward in a controlled manner while performing the Nordic hamstring exercise and to generate maximal eccentric force with the knee flexor muscles throughout the descent phase. Verbal cues were provided during the lowering phase to encourage maintenance of the highest possible eccentric tension in the target muscle group. In the event of pain, discomfort, or technical non-compliance during testing, the supervising researcher implemented the necessary adjustments to ensure the safe continuation of the measurement procedure [25]. Each player performed three maximal Nordic hamstring trials during each testing session, and the highest valid force value obtained from these trials was retained for statistical analysis. Prior to maximal testing, participants performed submaximal familiarization repetitions to ensure correct execution of the movement.

### 2.7. Training Design

The training program was designed to promote controlled and progressive development of eccentric hamstring muscle strength. Training intensity was categorized into three loading zones based on individual maximal eccentric force capacity: submaximal (60–70%), moderate-to-high (70–85%), and high intensity (≥85%). Across all exercises, the eccentric phase duration was standardized to 3–5 s, and participants were instructed to perform each repetition in a controlled manner to ensure consistency of mechanical loading. Training intensity was monitored using the Borg CR-10 Rating of Perceived Exertion (RPE) scale, with a target range set between 6 and 9. Repetitions reported with an RPE value ≥9 was excluded from analysis to prevent excessive loading and to maintain protocol standardization. Training sessions were conducted twice weekly, with a minimum recovery period of 48 h between sessions. All exercises were performed in the same predetermined order in each session. Throughout the training period, all sessions were supervised by the same qualified coach. Athletes with a participation rate below 85% were excluded from the analyses. Details of the training protocol and exercise-specific muscle targeting are provided in Table 3 and Table 4.

In addition to the Nordic hamstring exercise, supplementary eccentric exercises such as eccentric Romanian deadlifts and slide-based hamstring variations were incorporated into the program. External loading for these exercises was progressively increased throughout the training period to maintain the targeted intensity zones and ensure consistent overload of the hamstring musculature.

### 2.8. Training Compliance and Documentation

Participants’ adherence to the prescribed training program was systematically monitored and recorded by the researchers throughout the study period. Compliance criteria were defined as participation in at least 85% of the scheduled training sessions and completion of the sessions in accordance with the predefined set, repetition, tempo, and intensity parameters. Participants who were unable to complete the prescribed exercises due to injury or pain during the intervention period were excluded from the statistical analyses in order to preserve data integrity and analytical validity. All training sessions were supervised by the same strength and conditioning specialist throughout the study, a strategy adopted to minimize practitioner-related variability and ensure consistency in program implementation.

### 2.9. In-Session Load and Technical Monitoring

Training load and technical execution were systematically controlled using a multidimensional monitoring approach during all sessions. The duration of eccentric contraction phases was regulated using a metronome (60 beats·min^−1^) and verbal cues. Technical execution was continuously monitored through visual observation, with particular attention to pelvic positioning, trunk alignment, and knee joint angles. Training intensity was recorded after each set using the Borg CR-10 Rating of Perceived Exertion scale. When perceived exertion exceeded the target range (CR-10: 6–9), the corresponding set was terminated, or the load was individually adjusted to prevent excessive fatigue and technical deterioration.

### 2.10. Load Progression and Between-Team Standardization

Load progression was pre-planned according to weekly periods and standardized across all participants. Set and repetition volumes were progressively increased, while eccentric contraction duration was consistently maintained within a 3–5 s range across all exercises. Training intensity was individually adjusted based on perceived exertion levels recorded at the end of each session, ensuring that loading remained within the targeted intensity zones. In players exhibiting signs of excessive strain, the training load for the subsequent session was reduced by 10%, thereby supporting safe and sustainable progression.

The same training protocol was applied to both teams included in the study, and measurement timing and data recording procedures were implemented consistently across teams. This standardized approach ensured that any observed between-team differences reflected temporal performance changes rather than differences in training implementation.

### 2.11. Statistical Analysis

Data were analyzed using IBM SPSS Statistics software (version 26). To examine the effects of time (three measurement points) and team (Team A: Antalyaspor; Team B: Ankaragücü), a two-way mixed-design repeated-measures analysis of variance (mixed-design repeated-measures ANOVA) was conducted. The assumption of sphericity was assessed using Mauchly’s test, and Greenhouse–Geisser corrections were applied where necessary. Effect sizes were reported as partial eta squared (η^2^_p_), and Bonferroni-adjusted post hoc tests were used for multiple comparisons. The level of statistical significance was set at *p* < 0.05. Normality of distribution, homogeneity of variances, and potential outliers were examined prior to conducting the ANOVA analyses to verify the underlying statistical assumptions.

## 3. Results

Given the multidimensional nature of eccentric hamstring performance, results are presented across multiple mechanical metrics to allow a comprehensive, parameter-specific interpretation of adaptation patterns.

Mixed-design repeated-measures ANOVA results: Time effect, F (2, 76) = 0.82, *p* = 0.443, η^2^_p_ = 0.021; Team effect, F (1, 38) = 14.67, *p* < 0.001, η^2^_p_ = 0.278; Time × Team interaction, F (2, 76) = 3.87, *p* = 0.025, η^2^_p_ = 0.092. Bonferroni-adjusted pairwise comparisons indicated that the observed interaction was mainly driven by an increase in left maximal hamstring force in Team B from pre-test to post-test, whereas Team A demonstrated a different temporal pattern across the measurement periods (Table 5).

Mixed-design repeated-measures ANOVA results: Time effect, F (2, 76) = 0.60, *p* = 0.554, η^2^_p_ = 0.015; Time × Team interaction, F (2, 76) = 3.29, *p* = 0.043, η^2^_p_ = 0.080; Team effect, F (1, 38) = 0.28, *p* = 0.601, η^2^_p_ = 0.007. Bonferroni-adjusted pairwise comparisons indicated that the significant interaction reflected different temporal change patterns in right maximal hamstring force between the two teams across the measurement periods (Table 6).

Mixed-design repeated-measures ANOVA results: Time effect, F (2, 76) = 8.54, *p* < 0.001, η^2^_p_ = 0.183; Time × Team interaction, F (2, 76) = 0.41, *p* = 0.666, η^2^_p_ = 0.011; Team effect, F (1, 38) = 2.55, *p* = 0.119, η^2^_p_ = 0.063 (Table 7).

Mixed-design repeated-measures ANOVA results: Time effect, *F* (2, 76) = 19.82, *p* < 0.001, η^2^_p_ = 0.343; Time × Team interaction, *F* (2, 76) = 0.86, *p* = 0.428, η^2^_p_ = 0.022; Team effect, *F* (1, 38) = 78.86, *p* < 0.001, η^2^_p_ = 0.675 (Table 8).

Mixed-design repeated-measures ANOVA results: Time effect, *F* (2, 76) = 10.46, *p* < 0.001, η^2^_p_ = 0.216; Time × Team interaction, *F* (2, 76) = 1.43, *p* = 0.245, η^2^_p_ = 0.036; Team effect, *F* (1, 38) = 0.20, *p* = 0.658, η^2^_p_ = 0.005 (Table 9).

Mixed-design repeated-measures ANOVA results: Time effect, *F* (2, 76) = 18.45, *p* < 0.001, η^2^_p_ = 0.327; Time × Team interaction, *F* (2, 76) = 3.80, *p* = 0.027, η^2^_p_ = 0.091; Team effect, *F* (1, 38) = 8.56, *p* = 0.006, η^2^_p_ = 0.184. Bonferroni-adjusted pairwise comparisons indicated that the significant interaction reflected different temporal adaptation patterns in left average hamstring force between the two teams across the measurement periods (Table 10).

Mixed-design repeated-measures ANOVA results: Time effect, F (2, 76) = 13.59, *p* < 0.001, η^2^_p_ = 0.263; Time × Team interaction, F (2, 76) = 3.51, *p* = 0.035, η^2^_p_ = 0.085; Team effect, F (1, 38) = 9.81, *p* = 0.003, η^2^_p_ = 0.205. Bonferroni-adjusted pairwise comparisons indicated that the significant interaction reflected different temporal adaptation patterns in right average hamstring force between the two teams across the measurement periods (Table 11).

Mixed-design repeated-measures ANOVA results: Time effect, *F* (2, 76) = 17.60, *p* < 0.001, η^2^_p_ = 0.317; Time × Team interaction, *F* (2, 76) = 0.41, *p* = 0.663, η^2^_p_ = 0.011; Team effect, *F* (1, 38) = 3.02, *p* = 0.090, η^2^_p_ = 0.074 (Table 12).

Mixed-design repeated-measures ANOVA results: Time effect, F (2, 76) = 43.95, *p* < 0.001, η^2^_p_ = 0.536; Time × Team interaction, F (2, 76) = 38.58, *p* < 0.001, η^2^_p_ = 0.504; Team effect, F (1, 38) = 1.60, *p* = 0.214, η^2^_p_ = 0.040. Bonferroni-adjusted pairwise comparisons indicated that the significant interaction reflected different temporal adaptation patterns in left maximal hamstring impulse between the two teams across the measurement periods (Table 13).

Mixed-design repeated-measures ANOVA results: Time effect, F (2, 76) = 17.72, *p* < 0.001, η^2^_p_ = 0.318; Time × Team interaction, F (2, 76) = 4.02, *p* = 0.022, η^2^_p_ = 0.096; Team effect, F (1, 38) = 4.06, *p* = 0.051, η^2^_p_ = 0.096. Bonferroni-adjusted pairwise comparisons indicated that the significant interaction reflected different temporal adaptation patterns in right maximal hamstring impulse between the two teams across the measurement periods (Table 14).

Mixed-design repeated-measures ANOVA results: Time effect, F (2, 76) = 0.98, *p* = 0.380, η^2^_p_ = 0.025; Time × Team interaction, F (2, 76) = 0.22, *p* = 0.802, η^2^_p_ = 0.006; Team effect, F (1, 38) = 17.16, *p* < 0.001, η^2^_p_ = 0.311 (Table 15).

## 4. Discussion

In this study, the effects of a football-specific eccentric hamstring training model implemented in professional football players on left and right maximal hamstring force (LFM, RMF), maximal hamstring torque (LMT, RMT), mean force values (LAF, RAF), maximal force asymmetry (MI), mean asymmetry (AI), left and right maximal impulse (LMI, RMI), and impulse asymmetry (IB) were examined with respect to time and Team factors. The findings indicate that different mechanical variables may exhibit distinct response profiles over the course of the intervention. MI and AI exhibited significant time-dependent changes, whereas no significant team or time × Team interaction effects were observed. Significant time-dependent increases were observed for LMT, RMT, LAF, and RAF, with Team main effects also reaching significance for LMT, LAF, and RAF. For impulse-based variables, both the main effect of time and the time × Team interaction were significant for LMI and RMI, whereas for IB, only the Team main effect was significant, with no significant time or interaction effects.

The time-dependent increases observed bilaterally in maximal torque values appear consistent with stable adaptations in eccentric hamstring capacity. This finding aligns with existing literature indicating that eccentric loading can influence the muscle length–tension relationship and enhance force production capacity at longer muscle lengths [26,27]. Reports of increases in fascicle length following eccentric training, particularly in the biceps femoris long head, together with sarcomere-level remodeling and concurrent improvements in eccentric knee flexor strength [28,29,30], provide plausible mechanistic explanations for the present findings. Furthermore, evidence demonstrating that eccentric protocols with differing volumes and implementation characteristics can elicit comparable architectural and strength adaptations suggests that these mechanisms may emerge independently of a highly specific loading context [31]. However, as no direct assessments of muscle architecture or sarcomere adaptations were conducted in the present study, such structural adaptations should be interpreted as hypothetical mechanistic explanations rather than as causal inferences.

Within a multiscale adaptation framework, time-dependent changes observed in eccentric force outcomes may suggest that strength responses are reflected differently depending on the mechanical metric employed. In this context, maximal eccentric hamstring strength did not demonstrate a pronounced time-dependent increase when all athletes were considered collectively; however, team-level responses showed differentiation, underscoring the context-dependent nature of peak force adaptations. Previous studies have reported increases in eccentric knee flexor strength following Nordic hamstring-based interventions [29], although the magnitude of these responses appears to vary according to athlete profile, baseline performance level, and loading organization [28,32]. Eccentric knee flexor strength is therefore influenced by factors such as fatigue state, temporal placement of training, and load management [33,34]. These findings suggest that maximal force measurements alone may be insufficient to capture key mechanical characteristics of eccentric function. Mean eccentric force, by contrast, reflects the continuity of force production across the phase and provides a phase-sensitive representation of mechanical loading. Force–time-based metrics such as mean force and impulse may therefore offer more functionally relevant indicators that complement peak force outputs in the evaluation of eccentric adaptations. Although field-based NordBord assessments demonstrate high reliability, the sensitivity of absolute peak force changes and interlimb differences may still be influenced by baseline strength levels and individual response variability [22,32].

By contrast, impulse-related variables exhibited the clearest and most consistent adaptation patterns across time and teams in the present study. Because impulse reflects the temporal integration of force produced throughout the eccentric phase, it represents a mechanical dimension of performance that is distinct from peak force values [35]. This finding suggests that eccentric adaptations are not confined solely to maximal force magnitude but may also manifest in the capacity to sustain force production across the phase. In this regard, impulse metrics may provide additional insight into eccentric hamstring performance characteristics beyond peak and mean force measures, thereby enabling a more comprehensive evaluation of eccentric hamstring function [36,37]. Moreover, impulse can be considered a functionally relevant performance indicator in field settings, as it reflects the mechanical tolerance of the muscle–tendon unit to eccentric loading and its capacity to sustain controlled force production.

In the present study, the time-dependent changes observed in maximal force asymmetry, together with the similarity of these changes between teams, suggest that interlimb force distribution is responsive to eccentric loading. Nevertheless, interpretation of these changes within an injury-related context warrants caution. Hamstring strain injury (HSI) risk is recognized as a multifactorial construct, and it has been reported that a single strength indicator, including interlimb asymmetry, is insufficient to explain injury risk in isolation [38]. Athletes with a history of HSI have been shown to exhibit lower eccentric knee flexor strength, with interlimb force differences potentially becoming persistent over time [16,29]. As participants in the present study were not stratified according to previous hamstring strain injury history, changes in interlimb asymmetry are more appropriately interpreted as a mechanical component of the neuromuscular performance profile rather than as direct clinical risk indicators.

Methodological limitations related to measurement approaches also play a decisive role in the interpretation of asymmetry variables. In NordBord-based assessments, both the magnitude and direction of interlimb asymmetry have been reported to fluctuate over short time intervals [22], and incomplete standardization of parameter definitions and calculation methods in field-based systems may further limit the comparability of findings across studies [39]. The literature emphasizes that Minimal Detectable Change (MDC) thresholds should be considered to determine whether observed changes reflect true performance adaptations, as distinguishing between natural biological variability and genuine adaptation can be challenging for certain metrics [40,41]. Accordingly, while asymmetry-related findings are informative for characterizing the mechanical profile of eccentric performance, their use as definitive decision-making thresholds carries methodological constraints and contextual risk.

At a theoretical level, it has been proposed that mechanical gains observed following eccentric loading may be associated with the muscle’s length–tension behavior and history-dependent force production mechanisms [42,43,44]. Although these mechanisms are primarily derived from hypotheses tested under controlled experimental settings, they provide a conceptual framework for understanding the physiological underpinnings of the eccentric phase. As such relationships cannot be directly verified through field-based measurements, further studies incorporating advanced imaging techniques and micromechanical assessments are warranted. Accordingly, the present findings should be interpreted as descriptive indicators of adaptation reflecting changes in the mechanical profile of eccentric performance, while causal inferences regarding underlying mechanisms remain beyond the scope of this study.

### 4.1. Limitations

The present findings should be interpreted within the context of several methodological limitations. The inclusion of professional football players competing at the same league level restricts the generalizability of the results to different age groups, performance levels, and sporting disciplines. Baseline differences in strength profiles between teams suggest that the observed Team × time interactions may be influenced not only by the intervention itself but also by pre-existing neuromuscular differences. An additional limitation of the present study is the absence of a non-intervention control group, which limits causal inference regarding the effectiveness of the eccentric hamstring training program. The absence of direct assessments of muscle architecture, fascicle length, and sarcomere-level adaptations limits interpretation of changes in eccentric force and torque to indirect mechanistic inferences. Moreover, the lack of control over in-season training loads, external stressors, and prior eccentric exposure, together with the inherent variability associated with field-applied NordBord assessments, may complicate interpretation of small changes, particularly in impulse- and asymmetry-related variables. Multiple statistical tests were conducted across several mechanical variables, which may increase the risk of Type I error. Accordingly, the results should be viewed not as evidence of causal mechanisms, but as descriptive findings characterizing the mechanical profile of eccentric performance.

### 4.2. Practical Applications

Field-based assessments of eccentric hamstring performance such as those obtained using the NordBord system, may serve as a complementary tool for strength and performance practitioners in monitoring in-season eccentric hamstring capacity. Given that evaluating eccentric function solely through peak force may provide a limited perspective, the concurrent monitoring of phase-based metrics such as mean force and impulse may allow for a more comprehensive interpretation of training-induced adaptations. Rather than focusing on absolute values alone, interpreting these changes in relation to an athlete’s baseline profile, period-specific loading context, and individual response characteristics may render within-athlete longitudinal monitoring more informative than between-athlete comparisons.

Indicators of interlimb asymmetry may not be appropriate to use as definitive decision-making thresholds and are better interpreted as a contextual component of a broader neuromuscular performance profile. As injury incidence was not monitored in the present study, eccentric force and asymmetry outputs may be less informative when interpreted in isolation for injury risk assessment and are better considered within a multifactorial monitoring framework.

## 5. Conclusions

This study provides evidence that within the context of a field-based eccentric hamstring training program, different mechanical force outputs may exhibit distinct response profiles over time. The findings suggest that eccentric hamstring function cannot be fully represented by maximal force magnitude alone, and that phase-based force–time characteristics may constitute complementary components of the adaptation profile. These results support the interpretation that eccentric adaptations may emerge within a metric-dependent mechanical profile rather than as a uniform response pattern. Moreover, the divergent change patterns observed in interlimb asymmetry variables indicate that asymmetry may represent a dynamic characteristic sensitive to the specific mechanical context being assessed, rather than a fixed trait. Collectively, these findings align with the growing emphasis on multi-metric approaches in the literature and support the notion that evaluating eccentric hamstring function at the level of its mechanical profile may offer a more holistic conceptual framework.

## Figures and Tables

**Table 1:** Descriptive Characteristics of the Participants by Team.

	Antalyaspor (*n*: 20)	Ankaragücü (*n*: 20)
Age (Years)	20.62 ± 0.23	21.23 ± 0.44
Height (cm)	179.16 ± 6.95	176.04 ± 7.08
Training experience (years)	8.55 ± 0.76	8.45 ± 0.69

**Table 2:** Standardized Dynamic Warm-Up Protocol Applied Prior to Testing.

Phase	Duration	Exercise Type	Exercises	Volume/ Parameters	Primary Purpose
Phase 1: Joint Mobilization	2–3 min	Dynamic joint mobilization	Hip flexion/extension and abduction/adduction	10 repetitions per movement	Increase joint range of motion and promote synovial fluid circulation
			Knee flexion/extension	10 repetitions	Prepare knee joint for mechanical loading
			Ankle dorsiflexion/plantarflexion and inversion/eversion	10 repetitions	Enhance ankle joint mobility and stability
Phase 2: Dynamic Stretching	2–3 min	Dynamic stretching	Dynamic hamstring stretch (leg swings)	10 repetitions per leg	Increase muscle elasticity and reduce passive stiffness
			Walking knee hugs	10 steps	Activate posterior chain and improve hip mobility
			Walking lunges (glute-focused)	8–10 steps	Prepare gluteal muscles for activation demands
Phase 3: Muscle Activation	2–4 min	Low-to-moderate intensity neuromuscular activation	Glute bridge	2 × 8–10 reps (2 s eccentric/1 s concentric)	Enhance gluteal activation and pelvic stability
			Standing hamstring curl	2 × 8–10 reps (controlled tempo)	Increase hamstring neuromuscular readiness
			Nordic hamstring (submaximal familiarization)	1 × 3 reps (non-maximal)	Prime eccentric hamstring activation without inducing fatigue

The durations indicate approximate application intervals. All movements were performed at a controlled tempo and within a pain-free range of motion. At this stage, the Nordic hamstring exercise was applied solely for submaximal familiarization purposes and loading that could induce pre-test fatigue was deliberately avoided.

**Table 3:** Eccentric Hamstring Training Protocol.

Phase	Weeks	Exercise	Sets	Repetitions	Tempo (s)	Intensity (%MVC)	Intensity (CR-10)	Volume (Reps/Session)	Rest (s)
Adaptation	1–3	Nordic Hamstring	2	5	0–5	60–70%	6–7	10	90–120
		Eccentric Hamstring Slide		6	0–4			12	90–120
		Single-Leg Eccentric RDL		6	0–4			12	90–120
Progressive Loading	4–7	Nordic Hamstring	3	6	0–5	70–85%	7–8	18	90
		Eccentric Hamstring Slide		8	0–4			24	90
		Single-Leg Eccentric RDL		8	0–4			24	90
Maximal Loading	8–10	Nordic Hamstring	3	8	0–5	≥85%	8–9	24	60–90
		Eccentric Hamstring Slide		10	0–4			30	60–90
		Single-Leg Eccentric RDL		10	0–4			30	60–90

MVC% = percentage of maximal voluntary contraction. CR-10 = Borg Rating of Perceived Exertion Scale. Tempo values indicate the duration of the eccentric phase (s). Volume represents the total number of repetitions per exercise. Load progression was planned on a weekly basis, and in cases where signs of excessive fatigue were observed, the load was reduced by 10% in the subsequent session.

**Table 4:** Exercise-Specific Targeting of Hamstring Muscle Heads.

Exercise	Primary Target	Secondary Target(s)	Functional Emphasis
Nordic Hamstring	Biceps Femoris (Long Head)	Semitendinosus	Knee-dominant eccentric strength
Eccentric Hamstring Slide	Semitendinosus	Semimembranosus	Combined hip–knee eccentric control
Single-Leg Eccentric RDL	Semimembranosus	Biceps Femoris (Long Head)	Hip-dominant eccentric strength and unilateral control

Primary and secondary target muscle heads were classified based on EMG activation patterns and mechanical loading characteristics reported in the literature. Potential overlap in muscle activation across exercises should be taken into consideration.

**Table 5:** Temporal changes in left maximal hamstring force (Mean ± SD) for the two teams.

	Pre	Mid	Post
Team A	22.,065.25 ± 5861.37	22.836.25 ± 6438.89	20.141.50 ± 5667.04
Team B	23.227.95 ± 2269.60	25.209.50 ± 4176.73	27.144.25 ± 4271.16

**Note**: Left maximal hamstring force (LFM) values are expressed in Newtons (N).

**Table 6:** Temporal changes in right maximal hamstring force (Mean ± SD) in the two teams.

	Pre	Mid	Post
Team A	19.137.50 ± 1807.29	19.675.25 ± 1879.96	20.513.00 ± 2093.92
Team B	20.419.50 ± 3279.92	21.084.00 ± 3277.93	19.145.50 ± 6251.07

**Note:** Right maximal hamstring force (RMF) values are expressed in Newtons (N).

**Table 7:** Temporal changes in maximal hamstring strength asymmetry (Mean ± SD) in the two teams.

	Pre	Mid	Post
Team A	3.40 ± 1.19	3.67 ± 1.65	3.71 ± 1.62
Team B	2.85 ± 0.91	3.00 ± 0.81	3.12 ± 0.84

**Note:** Maximal hamstring force asymmetry (MI) values are expressed as percentages (%).

**Table 8:** Temporal changes in left maximal hamstring torque (Mean ± SD) in the two teams.

	Pre	Mid	Post
Team A	1.81 ± 0.38	1.91 ± 0.41	2.06 ± 0.39
Team B	3.41 ± 0.76	3.49 ± 0.72	3.76 ± 0.80

**Note:** Left maximal hamstring torque (LMT) values are expressed in Newton-meters (N·m).

**Table 9:** Temporal changes in right maximal hamstring torque (Mean ± SD) in the two teams.

	Pre	Mid	Post
Team A	3.40 ± 0.77	3.52 ± 0.70	3.71 ± 0.56
Team B	3.37 ± 1.54	3.69 ± 1.53	4.04 ± 1.38

**Note:** Right maximal hamstring torque (RMT) values are expressed in Newton-meters (N·m).

**Table 10:** Temporal changes in left average hamstring force during the eccentric phase (Mean ± SD) in the two teams.

	Pre	Mid	Post
Team A	2.11 ± 0.80	2.44 ± 0.81	2.69 ± 0.99
Team B	3.09 ± 0.90	3.14 ± 0.87	3.32 ± 0.80

**Note:** Left average hamstring force during the eccentric phase (LAF) values are expressed in Newtons (N).

**Table 11:** Temporal changes in right average hamstring force during the eccentric phase (Mean ± SD) in the two teams.

	Pre	Mid	Post
Team A	2.84 ± 0.73	3.12 ± 0.50	3.34 ± 0.53
Team B	3.95 ± 1.24	4.12 ± 1.31	4.12 ± 1.31

**Note:** Right average hamstring force during the eccentric phase (RAF) values are expressed in Newtons (N).

**Table 12:** Temporal changes in average hamstring force asymmetry (Mean ± SD) in the two teams.

	Pre	Mid	Post
Team A	4.93 ± 2.00	5.16 ± 1.93	5.48 ± 1.96
Team B	5.95 ± 1.47	6.05 ± 1.53	6.38 ± 1.36

**Note:** Average hamstring force asymmetry (AI) values are expressed as percentages (%).

**Table 13:** Temporal changes in left maximal hamstring impulse (Mean ± SD) in the two teams.

	Pre	Mid	Post
Team A	5.03 ± 0.72	5.74 ± 0.65	6.39 ± 0.53
Team B	6.19 ± 1.58	6.06 ± 1.41	6.24 ± 1.47

**Note:** Left maximal hamstring impulse (LMI) values are expressed in Newton-seconds (N·s).

**Table 14:** Temporal changes in right maximal hamstring impulse (Mean ± SD) in the two teams.

	Pre	Mid	Post
Team A	6.20 ± 0.56	6.80 ± 0.63	6.85 ± 0.58
Team B	5.84 ± 1.23	5.94 ± 1.25	6.24 ± 1.40

**Note:** Right maximal hamstring impulse (RMI) values are expressed in Newton-seconds (N·s).

**Table 15:** Temporal changes in impulse asymmetry during the eccentric phase (Mean ± SD) in the two teams.

	Pre	Mid	Post
Team A	8.33 ± 0.67	8.35 ± 0.67	8.40 ± 0.93
Team B	6.90 ± 1.36	6.85 ± 1.38	6.95 ± 1.48

**Note:** Impulse asymmetry during the eccentric phase (IB) values are expressed as percentages (%).

## Data Availability

The raw data supporting the conclusions of this article will be made available by the authors on request.
